# δ(^18^O/^16^O) Determinations in Water Using Inductively Coupled Plasma–Tandem Mass Spectrometry

**DOI:** 10.1021/acs.analchem.5c02607

**Published:** 2025-09-16

**Authors:** Shaun T. Lancaster, Johanna Irrgeher, Remi Dallmayr, Elisa Conrad, Maria Hörhold, Pascal Bohleber, Melanie Behrens, Federica Camin, Klara Žagar, Polona Vreča, Thomas Prohaska

**Affiliations:** † Department of General, Analytical and Physical Chemistry, Chair of General and Analytical Chemistry, 27268 Montanuniversität Leoben, 8700 Leoben, Austria; ‡ Department of Physics and Astronomy, University of Calgary, Calgary, Alberta T2N 1N4, Canada; § 84597 Alfred Wegener Institute Helmholtz Centre for Polar and Marine Research (AWI), 27570 Bremerhaven, Germany; ∥ Terrestrial Environmental Radiochemistry Laboratory, Division of Physical and Chemical Sciences, 537042 Department of Nuclear Sciences and Applications, International Atomic Energy Agency, Vienna International Centre, 1400 Vienna, Austria; ⊥ Department of Environmental Sciences, Jožef Stefan Institute, 1000 Ljubljana, Slovenia

## Abstract

A fully validated measurement approach for the determination of oxygen isotope ratios in water using inductively coupled plasma–tandem mass spectrometry (ICP-MS/MS) is described for the first time. Deuterium was used as a reaction gas to mass-shift the oxygen isotopes to the OD_3_^+^ product ion, allowing for the removal of polyatomic ions (^16^O^1^H^+^ and ^16^O^1^H_2_^+^) generated in the plasma that interfere on the less abundant oxygen stable isotopes (^17^O and ^18^O). The developed methodology was validated for δ_VSMOW‑SLAP_(^18^O/^16^O) determinations using four internationally recognized IAEA reference materials, as well as 11 in-house laboratory isotope reference materials that were characterized by isotope ratio mass spectrometry and cavity ring-down spectroscopy. δ_VSMOW‑SLAP_(^18^O/^16^O) values determined by ICP-MS/MS and the reference methods overlapped within the uncertainties and showed no significant difference for water materials with a low matrix load. Elevated levels of sodium, chloride, and silicon in the matrix lead to isotope ratio shifts of up to 12‰ in both the positive and negative directions. Additionally, comparisons with indicative values from the reference materials demonstrated that δ_VSMOW‑SLAP_(^17^O/^16^O) determinations are also possible using the ICP-MS/MS approach. While the uncertainty of the developed approach (median uncertainty *u*_c_(δ_VSMOW‑SLAP_(^18^O/^16^O)) = 0.70‰) is not currently able to match that of existing methodologies, it is envisioned that oxygen isotope ratio determinations can be performed alongside multielemental analysis by a wider community for applications where lower uncertainties are not required.

## Graphical abstract

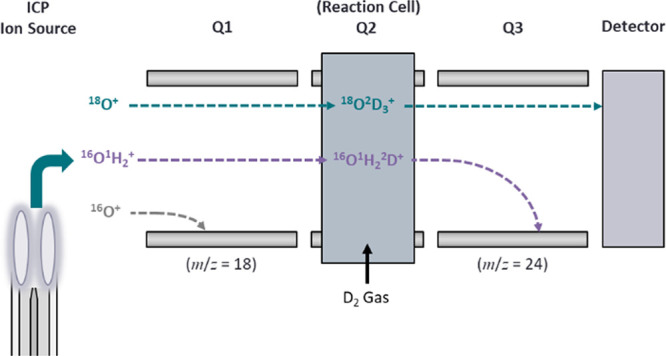

## Introduction

Stable isotope ratios of oxygen in water, in particular δ­(^18^O/^16^O), are of widespread interest in the fields of hydrology,^1–3^ climate research^4–6^ and food authentication.^7–9^ The differences in vapor pressure between the isotopologues of water lead to preferential evaporation of the lightest water molecule (^1^H_2_ ^16^O) and preferential condensation of the heaviest water molecule (^2^D_2_ ^18^O).^10^ The resulting fractionation effects lead to oxygen stable isotope ratio signatures in precipitation that can be used as a local and historical temperature proxy, for example in ice core research.^1,11,12^ Local^13,14^ and regional^15^ differences in oxygen isotope ratio signatures also allow for the tracing of geographical origin of waters, and has been used along with multielemental signatures in the testing of fraudulent mineral water,^16–19^ olive oil,^20^ dairy products,^21^ and wines.^22,23^ Stable isotope ratios of oxygen are most commonly expressed using the delta notation (δ)^24–26^ and expressed in per mil (‰), which for δ­(^18^O/^16^O) is calculated according to eq 1:

δsample,reference(O18/O16)=(O18O16sampleO18O16reference−1) 1

Isotope ratio mass spectrometry (IRMS) has for decades been the most widely used technique for oxygen isotope ratio determinations. The sample must be introduced in the gas phase, such as by dual inlet CO_2_–H_2_O equilibration method,^27^ or by pyrolysis with continuous flow.^28^ Alternatively, laser-based techniques, such as cavity ring-down spectroscopy (CRDS), have improved in the past decade, providing similar precisions to IRMS,^29–31^ although with a lower tolerance for matrix load. Both IRMS and CRDS generally provide precisions lower than 0.1‰.^32^

Quadrupole-based inductively coupled plasma mass spectrometry (ICP-MS) is a highly versatile and readily available technique that can be utilized for isotope ratio determinations and quantification of a vast number of elements on the periodic table. The technique is also compatible with many sample introduction approaches, such as aqueous sample aspiration, laser ablation and online-chromatographic separations. Moreover, unlike IRMS and CRDS, the ICP source does not require the generation of specific molecular species (e.g., CO, CO_2_, H_2_O) from the sample to operate, as all molecules are broken down to atoms that are subsequently ionized, offering a potentially simpler analytical approach. Thus, the ability to analyze stable oxygen isotope ratios by ICP-MS would be a great advantage to the community, as it could provide access to oxygen isotope ratio determinations in more diverse samples and applications. To date, however, successful determinations of oxygen isotope ratios have never been achieved using ICP-MS. The ICP ionization source is highly suited to elements with low ionization energies, i.e. metals. As such, the high ionization energy of oxygen of 13.61806 eV,^33^ results in notably lower ion formation, and thus low sensitivity. Additionally, since the plasma ionization source is operated under atmospheric conditions, this leads to high background signals due to the presence of atmospheric oxygen. Finally, numerous spectral interferences with mass-to-charge ratios (*m*/*z*) similar to that of the oxygen isotopes are formed in the plasma and require mass resolutions beyond the capability of a standard quadrupole-based ICP-MS to separate (Table S1). Of particular concern are the hydrogen-containing polyatomic ions of nitrogen (^15^N^1^H^+^ on *m*/*z* 16) and oxygen (^16^O^1^H_2_^+^ on *m*/*z* 18), as well as doubly charged sulfur ions (^32^S^2+^ on *m*/*z* 16) and argon ions (^36^Ar^2+^ on *m*/*z* 18). For these reasons, reliable determinations of oxygen isotopes by ICP-MS are challenging to obtain.

ICP–tandem mass spectrometry (MS/MS) utilizes a collision reaction cell (CRC) set between two quadrupoles; the mass filter and mass analyzer. The mass filter first applies a 1 amu bandpass to remove any ions that do not have the same nominal mass-to-charge ratio (*m*/*z*) as that of the target analyte. Second, the target analyte and remaining spectral interferences are separated in the CRC by applying a gas. Lastly, the mass analyzer applies a second 1 amu bandpass to allow only ions the nominal *m*/*z* of the target analyte to reach the detector. A wide variety of gases have been tested for typical ICP-MS/MS determinations.^34^ Reaction gases, such as ammonia^35^ (NH_3_), oxygen^35,36^ (O_2_) nitrous oxide^37,38^ (N_2_O), and hydrogen^39,40^ (H_2_), can be used to differentiate the target analyte and interferences; either by reacting only the target analyte to form a polyatomic ion that can be detected at a higher *m*/*z* (mass-shift determination), or by reacting only the interfering ion and measuring the target analyte at the same *m*/*z* as was introduced to the CRC (on-mass determination).

In this study, we present the first use of ICP-MS/MS to determine δ­(^18^O/^16^O) in water matrix, as well as preliminary data for the determination of δ­(^17^O/^16^O). H_2_ and deuterium (D_2_) reaction gases have been evaluated for the removal of interferences at *m*/*z* 16, 17, and 18. Following this, optimal operating conditions for the best signal to background ratio was achieved by varying the plasma power, and spray chamber temperature. The proposed ICP-MS/MS method was validated with the analysis of 4 water IAEA reference materials (RMs), as well as by comparison to in-house water laboratory reference materials (LRMs) with CRDS and IRMS.

## Methods and Materials

All preparations and measurements for ICP-MS/MS were made in a clean room (ISO class 8) with humidity controlled to 50–60%.

### Instrumentation

ICP-MS/MS measurements were carried out using a NexION 5000 (PerkinElmer, Waltham, MA, USA) equipped with a quadrupole-based dynamic reaction cell that has the ability to modify the applied bandpass through rejection parameter “a” (RPa) and “q” (RPq), which are related to the “a” and “q” parameters in the Mathieu equation for the stability of ions in a quadrupole.^41^ The orientation of the mass filter (Q1), reaction cell (Q2), and mass analyzer (Q3) for the example of interference removal on ^18^O^+^ is given in Figure 1. The instrument can facilitate up to four reaction cell gases, with one of the gas lines available for corrosive gas. In this study, the sample introduction system comprises of a peristaltic pump for sample uptake, a PFA MicroFlow nebulizer, and a SilQ cyclonic spray chamber. Instrumental operating parameters are provided in Table S2. Argon (Ar, purity 5.0 (≥99.999%); Linde Gas GmbH, Stadl-Paura, Austria) was used as the plasma gas, nebulizer gas, and auxiliary gas. Hydrogen (purity 5.0 (≥99.999%); Linde Gas GmbH) and deuterium (99.9% purity, 99.8% enrichment; Linde Gas GmbH) were evaluated as reaction gases for interference removal. The gases were delivered to the reaction cell using the instruments noncorrosive gas lines.

**Figure 1:**
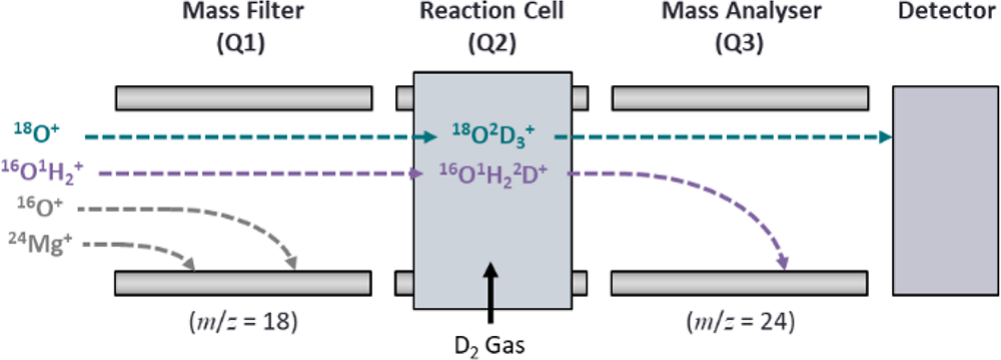
Diagram of the ICP-MS/MS setup used within this study using the example of mass-shift of ^18^O^+^ with deuterium gas.

IRMS and CRDS were used as reference analytical methods for the validation of the developed ICP-MS/MS approach. Further information regarding the instrumentation and methodology is provided in the Supporting Information (SI: Comparative analysis of oxygen isotope ratios in water by validated methodologies).

ICP-MS/MS and ion chromatography were used for multielemental analysis and anionic speciation to assess the matrix components of the materials used. The elements sodium, magnesium, aluminum, silicon, potassium, calcium, vanadium, chromium, manganese, iron, cobalt, nickel, copper, and zinc were analyzed by ICP-MS/MS, and fluoride, chloride, bromide, nitrate, nitride, and sulfate were analyzed by IC. Further information regarding the use of ICP-MS/MS and IC for the assessment of the matrix components of the materials used in this study is provided in the Supporting Information (SI: Methodology for multielement determinations; SI: Methodology for anion determinations).

### Chemicals, Standards, and (Certified) Reference Materials

A total of 11 in-house laboratory reference materials with a wide variation of δ­(^18^O/^16^O) values ranging from −54.03 to +0.4‰ were used within this study. Details about the materials and their reference δ_VSMOW‑SLAP_(^18^O/^16^O) values are provided in Table 1.

**Table 1:** List of In-House Laboratory Reference Materials and Internationally Recognized Reference Materials^43^ Used Within This Study

name	material description	reference δ_VSMOW‑SLAP_(^18^O/^16^O) (‰)	reference technique
VSMOW2	fresh water (RM)^44^	0 ± 0.02	IRMS
SLAP2	melted antarctic ice core (RM)^44^	–55.5 ± 0.02	IRMS
GRESP	melted snow (used as drinking water source) from Greenland (CRM)^45^	–33.40 ± 0.04	CRDS + IRMS
IAEA 604	deionized tap water combined with ^2^D-enriched water (CRM)^46^	–5.86 ± 0.04	CRDS + IRMS
LRM 1	distilled adriatic coastal seawater	+0.36 ± 0.04	IRMS
LRM 2	reagent grade I water prepared from tap water in Ljubljana (Reaktor Center), Slovenia	–9.12 ± 0.03	IRMS
LRM 3	melted snow water from the Kanin mountain, Slovenia	–18.91 ± 0.03	IRMS
LRM 4	melted snow water from Vostok, Antarctica	–54.03 ± 0.03	IRMS
LRM 5	distilled Mediterranean Sea water	+0.4 ± 0.03	CRDS
LRM 6	reagent grade I water prepared from tap water in Leoben, Austria	–10.77 ± 0.1	CRDS
LRM 7	reagent grade I water prepared from tap water in Leoben, Austria (spiked with enriched ^18^OH_2_)	–3.22 ± 0.1	CRDS
LRM 8	tap water from Bremerhaven, Germany	–7.34 ± 0.1	CRDS
LRM 9	mix of melted snow from polar regions	–26.64 ± 0.1	CRDS
LRM 10	surface snow from Antarctica (AWI in-house reference material)	–42.40 ± 0.02	CRDS
LRM 11	surface snow from Antarctica (AWI in-house reference material)	–53.01 ± 0.03	CRDS

Four water internationally recognized reference materials VSMOW2, SLAP2, GRESP, and IAEA 604 were analyzed (IAEA, Vienna, Austria). VSMOW2 and SLAP2 were used for calibration and normalization of the results to the VSMOW-SLAP scale.^42^ GRESP and IAEA 604 were used alongside in-house water laboratory reference materials for validation.

### ICP-MS/MS Analysis of Oxygen Isotope Ratios

#### Evaluation of ICP-MS/MS Cell Gases for Interference Removal on Oxygen Isotopes

Initial evaluation of cell gases for interference removal were carried out by disconnecting the sample introduction system at the nebulizer and capping the inlet so that only the background sources of oxygen from the atmospheric oxygen within the torch box and oxygen from impurities in the argon gas were analyzed. This approach was taken in order to avoid overloading the detector. The mass filter (Q1) was set to the nominal *m*/*z* of the target analyte (^16^O, ^17^O, or ^18^O) and the mass analyzer (Q3) was to scan from *m*/*z* 15–25. The mass-scans were performed at gas flow rates of 1 mL min^–1^ to identify the product ions generated in the gas cell. Further tests were carried out using gas flow rates of 0.1–1.5 mL min^–1^ to identify how the formation rate of the product ions vary with H_2_ and D_2_ gas flow rates.

#### Evaluation of the Signal to Background Ratio Using ICP-MS/MS

The spray chamber and the nebulizer were washed thoroughly with reagent grade I water and dried using nitrogen in order to remove traces of dilute nitric acid and water from any previous multielemental analysis. A deuterium gas flow of 1 mL min^–1^ was applied to the gas cell. The mass filter (Q1) was set to the nominal *m*/*z* of the target analyte (^16^O, ^17^O, or ^18^O) and the mass analyzer (Q3) set to analyze OD_3_^+^ product ion (+6 amu). In order to reduce the signal intensity for oxygen in water to be within the pulse mode region of the detector (<2 × 10^6^ cps), the reaction cell RPa value setting was utilized to reject ions. The RPa was tuned to obtain a signal of approximately 500,000 cps for each oxygen isotope in reagent grade I water (LRM6) at a plasma power of 1600 W and a spray chamber temperature of 50 °C. The plasma power was varied between 750–1600 W and the spray chamber temperature was varied between 5–70 °C. Following this, the spray chamber and nebulizer were dried thoroughly with nitrogen to remove any water from the instrument, and the measurements were repeated at the same RPa settings sample introduction system disconnected at the nebulizer and the sample inlet capped. Here, the “background” is considered to be measured intensities due to the atmospheric oxygen within the torch box and oxygen from impurities in the argon gas, and the “signal” is the measured intensity of oxygen when introducing LRM6.

#### ICP-MS/MS Analysis of Oxygen Isotope Ratios in Water Standards and Internationally Recognized Reference Materials

Oxygen isotope ratios were determined using a standard-sample bracketing approach, where reagent grade I water was used as the bracketing standard. The mass filter (Q1) was set to the nominal *m*/*z* of the target analyte (^16^O, ^17^O, or ^18^O) and the mass analyzer (Q3) set to analyze OD_3_^+^ product ion (+6 amu). The sample probe was cleaned manually with a dry clean-room paper towel when switching between samples in order to minimize contamination. A peristaltic pump with orange-green tubing (4.7 μL min^–1^ rpm^–1^) was used to introduce the samples to the instrument. At the beginning of each sample measurement, the peristaltic pump speed was increased to 100 rpm for 90 s to flush the spray chamber and remove the previous water sample from the system. Analysis was then conducted at a pump speed of 18 rpm. The total uptake volume was approximately 1 mL per measurement.

#### Oxygen Isotope Ratio Data Evaluation and Uncertainty Assessment

In total, 40,000 data points were obtained for each oxygen isotope at a dwell time of 2 ms and quadrupole settling time of 0.2 ms, giving a total acquisition time of 4 min 24 s per measurement. The first 10 000 data points were omitted to allow for the stabilization of the instrument after sample uptake (Figure S2). To improve the precision of the analysis, the following data reduction procedure was employed: A ratio was taken for each pair of data points obtained for ^18^O and ^16^O to obtain 30 000 measured ^18^O/^16^O data points. An outlier correction was then applied to remove any ^18^O/^16^O data points that were outside two times the standard deviation (95% confidence interval) of the full 30 000-point ^18^O/^16^O data set. The data was averaged as 2000 sweeps/replicate and 15 replicates, which were used to calculate the average measured isotope ratio and standard deviation. By performing the outlier correction, an average of 1350 data points (4.5%) were identified as outliers per measurement, and their removal improved the RSD of a measurement by an average of 13%. This same data reduction procedure was utilized for obtaining the measured ^17^O/^18^O values.

δ­(^17^O/^16^O) and δ­(^18^O/^16^O) were calculated relative to a working standard (WS), in this case LRM6 (Table 1), following eq 2:

δsample,WS(Oi/O16)=(OiO16sampleOiO16WS−1) 2

where *i* = 17 or 18. Calibrated isotope ratios were then calculated relative to the VSMOW-SLAP scale using eq 3 in accordance with the VSMOW2 and SLAP2 reference material certificate:^44^

$$\delta_{\mathrm{sample},\mathrm{cal}}{(}^{i}O{/}^{16}O)=\delta_{\mathrm{LS}1,\mathrm{cal}}{(}^{i}O{/}^{16}O)+\left(\delta_{\mathrm{sample},\mathrm{WS}}{(}^{i}O{/}^{16}O)-\delta_{\mathrm{LS}1,\mathrm{WS}}{(}^{i}O{/}^{16}O)\right)\times \frac{\left(\delta_{\mathrm{LS}2,\mathrm{cal}}{(}^{i}O{/}^{16}O)-\delta_{\mathrm{LS}1,\mathrm{cal}}{(}^{i}O{/}^{16}O)\right)}{\left(\delta_{\mathrm{LS}2,\mathrm{WS}}{(}^{i}O{/}^{16}O)-\delta_{\mathrm{LS}1,\mathrm{WS}}{(}^{i}O{/}^{16}O)\right)}$$ 3

where “cal” denotes calibrated measurements relative to the VSMOW-SLAP scale. LS1 and LS2 are laboratory standards used for calibration, in this case VSMOW2 and SLAP2 respectively.

Combined measurement uncertainties were calculated using a Kragten spreadsheet approach.^47^ This work primarily presents the uncertainty of repeat observations, where the intermediate precision of each measured delta value is calculated using the standard error of the mean ( $s/\sqrt{n}$ ) in accordance with the Guide to Uncertainty of Measurements.^48^ Only the uncertainties for the terms in eq 3 were considered in the combined uncertainty calculation, as other contributions, such as drift and homogeneity of the standards, were found to be negligible (<5% contribution). Evaluations of single measurement uncertainties have been provided in the Supporting Information (SI: Single measurement uncertainty assessment).

## Results and Discussion

### Evaluation of Cell Gases for Interference Removal

Preliminary testing was carried out ascertain if H_2_ gas was capable of resolving the interferences on the isotopes of ^16^O, ^17^O, and ^18^O. The ratios obtained were compared to the expected ratios, calculated based on terrestrial isotopic abundances^49^ (^18^O/^16^O: 0.00205; ^17^O/^16^O: 0.000381). To highlight the need for the removal of interferences, the oxygen isotopes were measured on-mass in the absence of a cell gas in the CRC, which provided isotope ratios far from the expected values (^18^O/^16^O: 4.04; ^17^O/^16^O: 0.0396).

H_2_ was applied at 1 mL min^–1^ and reacted with ^16^O^+^ to form three product ions: OH^+^ (+1 amu), OH_2_^+^ (+2 amu), and OH_3_^+^ (+3 amu) (Figure 2A). The OH_3_^+^ product ion provided the greatest signal intensity (formation rate of 48% compared to no gas). Varying the H_2_ gas flow rate caused the product ions to form at different rates, with the low-order product ion (OH^+^) forming preferentially lower H_2_ flow rates and the higher-order product ion (OH_3_^+^) forming at higher H_2_ flow rates (Figure S1). In theory, the OH_3_^+^ product ion would have the greatest potential for the removal of hydrogen-based interferences of ^16^O^+^ on ^17^O^+^ and ^18^O^+^, as these isotopes would be separated from their interferences by at least 1 amu following the mass-shift reaction (e.g., target analyte: ^18^O^+^ (*m*/*z* = 18) → ^18^O^1^H_3_^+^ (*m*/*z* = 21); interference: ^16^O^2^D^+^ (*m*/*z* = 18) → ^16^O^1^H_2_ ^2^D^+^ (*m*/*z* = 20)). In reality, however, significant interferences persisted, as the H_2_ gas used contains a natural abundance of the ^2^D isotope, which caused the interference to mass-shift an additional 1 amu to match the shifted-mass of the target analyte (e.g., ^16^O^2^D^+^ (*m*/*z* = 18) → ^16^O^1^H^2^D_2_^+^ (*m*/*z* = 21)) (Figure 2B,C). Furthermore, the level of interference from the ^2^D isotope appeared greater than expected, as natural H_2_ should contain only 0.0115% of the ^2^D isotope. It is possible that the hydrogen gas used within this study has a non-natural abundance of deuterium. However, D_2_ would be expected to react differently to H_2_ in the CRC due to their mass difference of 100%. Nevertheless, the preliminary isotope ratios obtained (^18^O/^16^O (as ^18^O^1^H_3_^+^/^16^O^1^H_3_^+^): 0.00778; ^17^O/^16^O (as ^17^O^1^H_3_^+^/^16^O^1^H_3_^+^): 0.00178) were greater than the expected ratios and H_2_ was not used further in this study.

**Figure 2:**
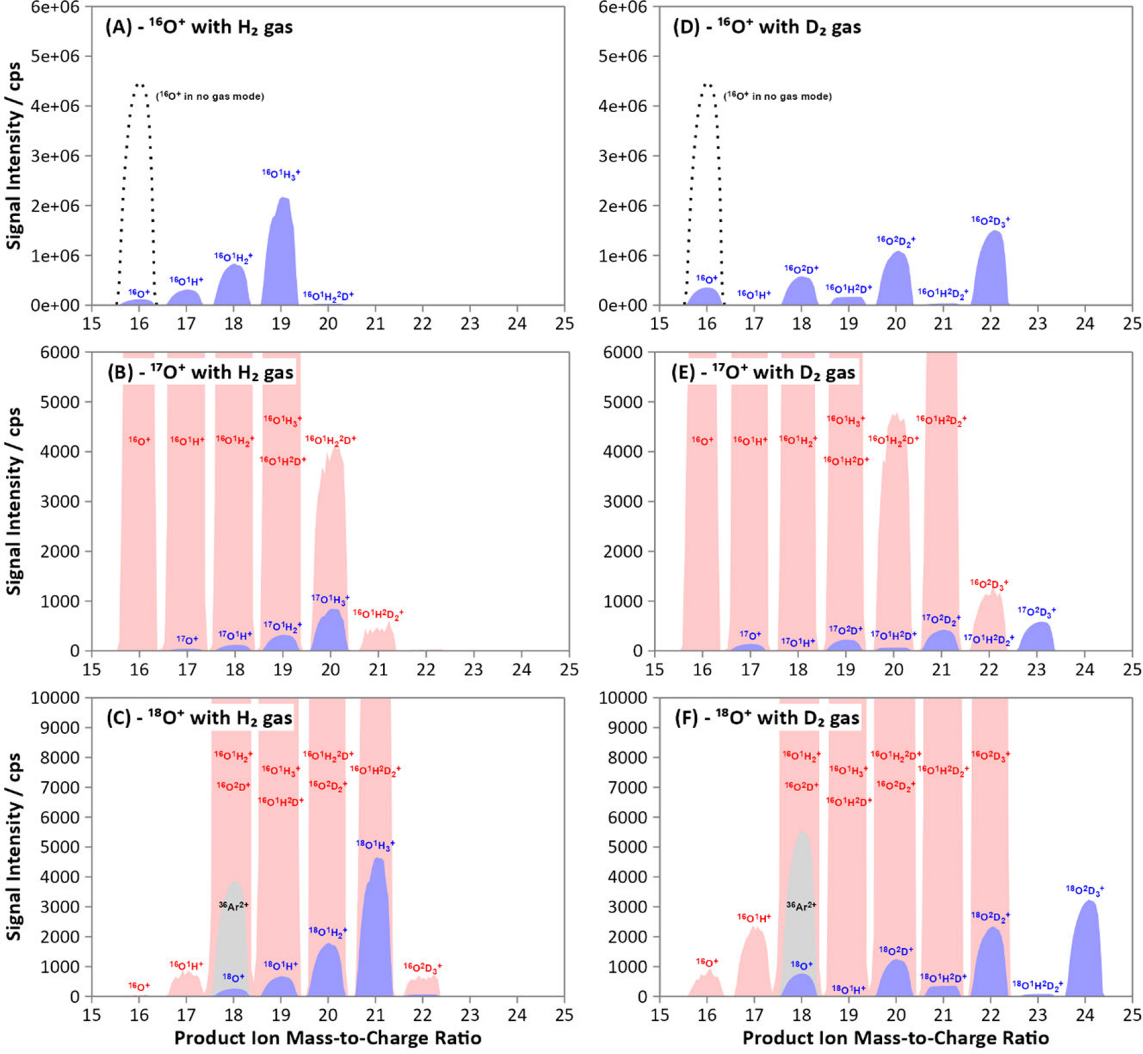
Product ion scans of stable oxygen isotopes with H_2_ (A-C) and D_2_ (D-F) cell gases at 1 mL min^–1^. The mass filter (Q1) was fixed at the nominal *m*/*z* of the target analyte (^16^O, ^17^O, ^18^O), and the mass analyzer (Q3) was set to scan between *m*/*z* 15–25. The product ion scans of ^16^O^+^ and ^40^Ar^2+^ have been overlaid on the product ion scans of the ^17^O^+^ and ^18^O^+^ isotopes to indicate the existence of remaining interferences. Product ion profiles of the target analyte are shown in blue, with the Ar- and ^16^O^+^-based interferences depicted in gray and red, respectively. The dotted line in (A) and (D) represents the signal of ^16^O^+^ in the absence of a cell gas.

To solve this issue, D_2_ was instead chosen as a reaction gas. The reaction of D_2_ with ^16^O^+^ was comparable to that of H_2_ (Figures 2D and S1), with the OD_3_^+^ product ion providing the greatest sensitivity (formation rate of 32% compared to no gas). The OD_3_^+^ product ion (+6 amu) was used for successful resolution of interferences (Figure 2E,F), as impurities of the ^1^H isotope in the cell gas can only cause the interferences to be shifted to lower masses that are further away from the shifted mass of the target analyte. As a result, the preliminary isotope ratios obtained (^18^O/^16^O (as ^18^O^2^D_3_^+^/^16^O^2^D_3_^+^): 0.00214; ^17^O/^16^O (as ^17^O^2^D_3_^+^/^16^O^2^D_3_^+^): 0.000411) were very close to that of the expected value. Therefore, D_2_ gas was used for all further developments of the method. It should be noted, though, that the resolution of interferences by this method relies on the mass filter within the MS/MS setup to remove ^23^Na^+^ and ^24^ Mg^+^ prior to mass-shifting with the CRC, and would likely not be replicated on single-quadrupole ICP-MS systems. This approach does, however, additionally remove interferences from doubly charged interferences of argon and sulfur, as these interferences do not form the ArD_6_^2+^ and SD_6_^2+^ required to interfere on the shifted mass of the target analytes.

Although the mass-shift approach could resolve interferences from OH^+^, there is a possibility that interferences could still arise from NH^+^. Separate product ion scans of ^14^N^+^ showed formation of the ^14^N^2^D_4_^+^ product ion, which indicates that ^14^N^2^D^+^ and ^15^N^1^H^+^ could be potential interferences for the reaction of ^16^O^+^ to ^16^O^2^D_3_^+^. In the context of this work, the expected interference is negligible as not only is the background of nitrogen in water low compared to oxygen, but the abundances of ^14^N^2^D^+^ and ^15^N^1^H^+^ are far lower than that of ^16^O^+^. However, for applications involving waters highly enriched deuterium, or for future applications to atmospheric δ­(^18^O/^16^O) measurements, interference from ^14^N^2^D^+^ on ^16^O^+^ have to be further investigated.

### Assessment of the Signal to Background Ratio

When introducing reagent grade I water (LRM6) into the ICP-MS/MS, the signal for ^16^O^+^ (measured as the ^16^O^2^D_3_^+^ product ion) was greater than the maximum range of the of the detector (approximately 1 × 10^9^ cps). Therefore, it was necessary to reduce the sensitivity of the instrument to bring the signal within a measurable range. Initial attempts were made by detuning the ion deflector situated after the sampling interface, that bends the ion path by 90° to deliver the positive ions to the mass spectrometer. A promising ^18^O/^16^O ratio (measured as ^18^O^2^D_3_^+^/^16^O^2^D_3_^+^) of 0.00228 was obtained, but with an RSD of 0.97% on the measured ratio (after data reduction of the signal had been applied). This was attributed to the detector requiring to switch between analog and pulse modes in order to capture the signals for both ^18^O^+^ and ^16^O^+^, respectively. Another approach was to constrict the bandpass of the quadrupole in the reaction cell by applying an RPa. As ICP-MS/MS is a single-collector instrument (i.e., each isotope is measured sequentially), the RPa is required to be specified for each measured oxygen isotope individually. It was observed that the magnitude of ions rejected for a given RPa value was not consistent between isotopes, likely due to slight discrepancies between the mass calibrations of the oxygen isotopes. Therefore, fractionation is unavoidable using this approach. However, since oxygen isotope ratios are typically calculated using the delta notation (e.g., δ­(^18^O/^16^O), eqs 1 and 2), any fractionation induced by applying RPa is accounted for because the delta notation only considers the relative difference in measured ratios obtained for the sample and the reference standard. This presented an additional opportunity, as the signal for each isotope of oxygen could be individually tuned to any intensity, since only the relative differences in measured isotope ratios are considered. Thus, the RPa was tuned using LRM6 until a signal of approximately 500 000 cps was reached for each oxygen isotope (as the OD_3_^+^ product ion) to have all isotopes of oxygen measured in the pulse region of the detector. Using this approach, markedly improved RSDs of 0.1–0.2% were obtained. Thus, the RPa approach was utilized in all subsequent developments.

Signal to background ratio optimizations were carried out using LRM6. Highly unstable oxygen signals were observed when using a spray chamber temperature of 5 °C, with average signal RSDs of 23% for all isotopes. Large dips in the signal were observed that appeared each time a drop of water was drained from the spray chamber via a peristaltic pump. Operating the spray chamber at temperatures higher than 20 °C resulted in the oxygen signals becoming much more stable, with the optimum RSD obtained at 50 °C (Figure 3). The large signal instability at lower temperatures correlated visually with the liquid draining from the spray chamber. Therefore, the improved stability at higher temperatures may be due a lower viscosity and surface tension of the water, leading to improved transfer of water through the spray chamber, with less buildup of liquid (due to condensation) at the drain. The signal to background ratio was not observed to change with the spray chamber temperature. Decreasing the plasma RF power from 1600 to 750 W caused an increase in sensitivity of oxygen from the water (Figure 4A), however it also increased the background oxygen signal. This increase could be explained by a corresponding decrease in the formation of polyatomic oxide species formed in the plasma, as was observed for ArO^+^ (measured on *m*/*z* 56). This resulted in a sharp decline of the signal to background ratio at lower plasma power (Figure 4B). For this study, the plasma RF power was operated at 1600 W, giving a signal to background ratio of approximately 16 000 for all oxygen isotopes.

**Figure 3:**
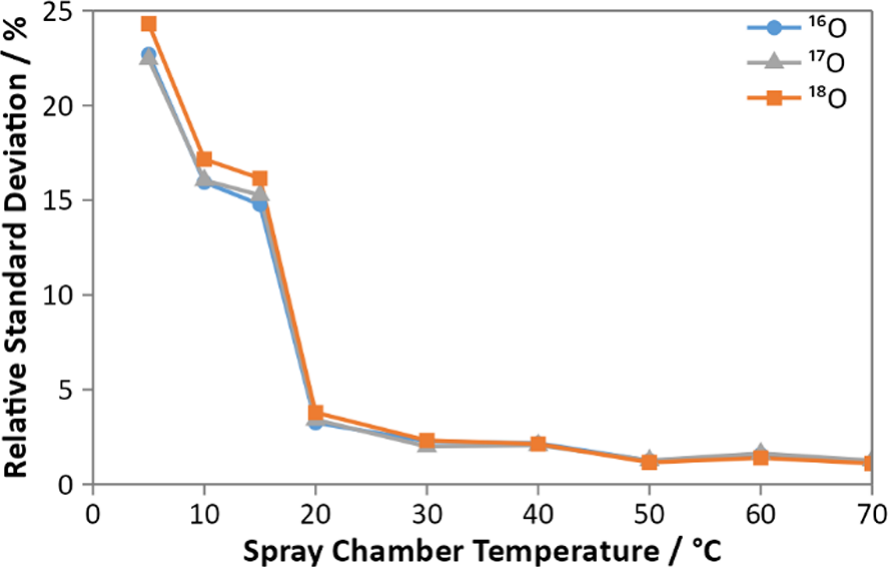
Variation of the obtained signal RSD with the temperature of the spray chamber for ^16^O (blue, circle), ^17^O (gray, triangle), and ^18^O (orange, square).

**Figure 4:**
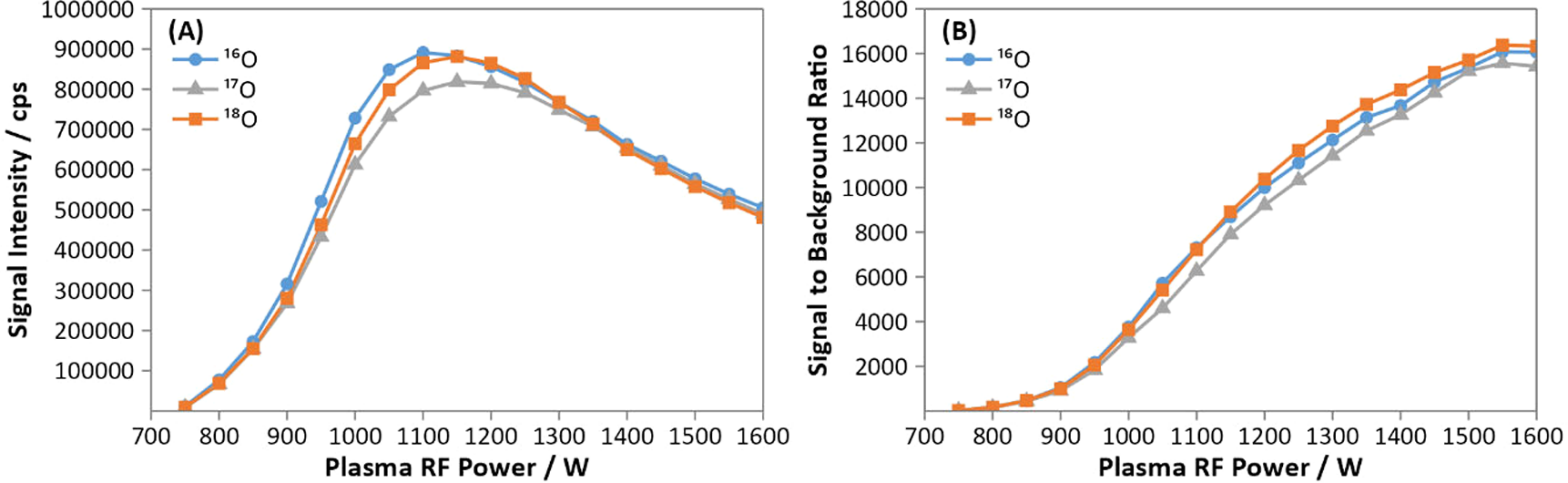
Variation of (A) the signal intensity, and (B) the signal to background ratio with the plasma RF power for ^16^O (blue, circle), ^17^O (gray, triangle), and ^18^O (orange, square).

### Method Validation

#### Determination of δ_VSMOW‑SLAP_(^18^O/^16^O)

The developed methodology was validated for δ­(^18^O/^16^O). Replicate measurements (*n* = 6) of the calibration materials by ICP-MS/MS gave a δ_VSMOW_(^18^O/^16^O) of −49.80 ± 0.58‰ for SLAP2 (reference δ_VSMOW‑SLAP_(^18^O/^16^O) value of −55.50 ± 0.02‰). Therefore, the normalization factor of 1.114 ± 0.013 (*u*_c_; *k* = 1) was applied to report the measured values on the VSMOW-SLAP scale. The intermediate precision (*k* = 1) for VSMOW2 and SLAP2 was 0.50 and 0.28‰ respectively.

Replicate measurements (*n* = 3) of the water LRMs analyzed by the developed ICP-MS/MS method were compared to the results obtained by CRDS and IRMS as reference techniques (Figure 5; Table S3). The line of best fit indicates an excellent correlation between the measurements of the proposed ICP-MS/MS methodology and the reference methods. The obtained slope of 1.004 was found to be not significantly different to the expected value of 1 within a 95% confidence limit (2-tailed *t* test; p = 0.82; standard error of the slope = 0.017). Additionally, the intercept was not found to be significantly different to the expected value of 0 within a 95% confidence limit (2-tailed *t* test; p = 0.18; standard error of the intercept = 0.51), indicating no significant systematic bias with the ICP-MS/MS approach.

**Figure 5:**
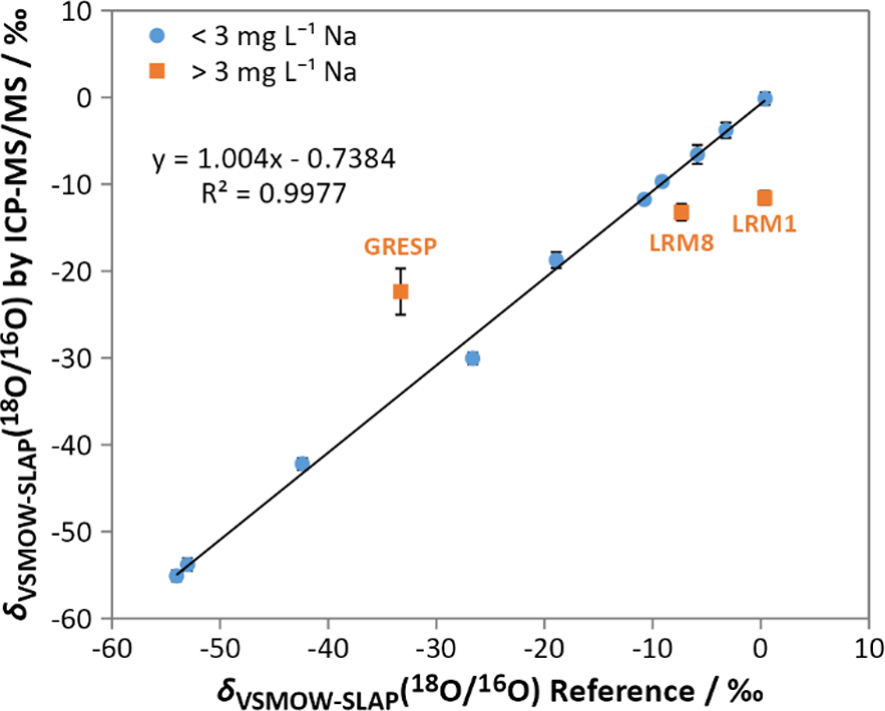
Comparison of δ_VSMOW‑SLAP_(^18^O/^16^O) values obtained for in-house standards by ICP-MS/MS (*n* = 3) with reference methods (IRMS, CRDS, and certified reference materials). Samples that contained elevated levels of sodium, silicon, and chloride were treated as outliers (orange squares, labeled) and were not included in the trendline. Error bars represent the combined measurement uncertainty (*k* = 1).

While the method has been demonstrated to work well, three clear outliers were however observed (Figure 5; orange squares), namely LRM1, LRM8, and the IAEA reference material GRESP. Further analysis of the matrix composition of the measured standards highlighted that these particular outliers contained a notably higher ionic matrix than the other standards, particularly regarding their sodium (4170–18 400 ng mL^–1^), silicon (GRESP: 20 300; LRM8:89 600 ng mL^–1^), and chloride (LRM1:130 000 ng mL^–1^) concentrations (Tables S4 and S5). This could potentially be explained by changes in ionization efficiency of oxygen in the plasma due to matrix suppression and enhancement effects. Elements with very low ionization energies that are highly abundant in natural water matrices (namely sodium (5.14 eV), magnesium (7.64 eV), calcium (6.11 eV)) cause suppression due to space-charge effects (repulsion between positive ions), with stronger effects observed the lower the ionization energy of the matrix element.^50^ These effects induce a mass-bias, with the lighter isotopes being suppressed more and thus transmission of heavier isotopes are favored,^51^ leading here to higher δ_VSMOW‑SLAP_(^18^O/^16^O) values (positive bias). Conversely, matrix elements with high ionization energies, such as carbon (11.26 eV) have been reported to cause enhancement effects by the process of charge transfer reactions, where the charge is transferred from the matrix element to the target analyte. This effect has been observed to be strongest for hard-to-ionize elements with excited states that have an energy similar to the ionization energy to carbon.^52,53^ Lighter isotopes are enhanced more than heavier isotopes and thus the transmission of lighter isotopes is favored, leading here to lower δ_VSMOW‑SLAP_(^18^O/^16^O) values (negative bias). However, carbon has a lower ionization energy than oxygen (13.62 eV), meaning such charge transfer reactions would not be expected to take place. It has previously been demonstrated that elements with low-lying metastable ionic states, such as silicon, phosphorus, sulfur, and chlorine are formed in the plasma and are available for charge transfer reactions.^37,54^ The energy of the metastable ionic states of these elements are close to the ionization energy of oxygen (Table S6). Another possible explanation for a negative bias could be due to different aerosol and transport processes. Given that the outliers contained varied levels of sodium, silicon, and chloride, it is likely that both suppressive and enhancement effects are occurring and the magnitude and direction of mass-bias is dependent on the ratio of these effects. A comprehensive follow-up study is required to assess the matrix effects for δ_VSMOW‑SLAP_(^18^O/^16^O) determinations in future. Moreover, strategies to overcome matrix effects are required to improve the robustness of the method for samples with elevated matrix components. One possible approach is to remove the matrix through solid-phase extraction methods, which is a typical approach for isotope ratio determinations by ICP-based methods. Alternatively, it may be possible to matrix-match by adding a salt at elevated concentrations to overcome slight differences in the matrix between samples and reference standards.

The obtained combined uncertainties (*k* = 1) for replicate determinations of samples with low matrix load ranged from 0.48 to 1.09‰, with a median uncertainty of 0.70‰. The three outliers with higher matrix load gave combined uncertainties of 0.79 to 2.66‰. However, there are too few samples with higher matrix load in this study to conclusively infer that the higher matrix levels lead to higher uncertainties, and this will need to be investigated further. In all cases, the highest contributions to the uncertainty budget originated from the replicate measurements of the samples and of the VSMOW2 calibration standard. Although the uncertainties for the ICP-MS/MS approach are an order of magnitude higher than that of the reference methods (Table 1), it is still low enough to be useful for distinguishing differences in water samples that have a wider spread of δ­(^18^O/^16^O) values, such as for climate studies using ice cores, or in polar precipitation. Further optimizations could be investigated in future in order to reduce the uncertainty further.

#### Determination of δ_VSMOW‑SLAP_(^17^O/^16^O)

In general, determinations of δ­(^17^O/^16^O) are much less routinely performed than for δ­(^18^O/^16^O). Nevertheless, the use of deuterium gas was noted to remove the interferences on *m*/*z* 17 and therefore the determination of δ­(^17^O/^16^O) may be possible by the ICP-MS/MS technique. Although δ­(^17^O/^16^O) determinations were not performed for the LRMs using the reference techniques in this study, indicative values are provided for the two additionally measured reference materials (GRESP and IAEA 604), as well as for the calibration materials (VSMOW2 and SLAP2).

Measurements of the calibration materials by ICP-MS/MS gave a δ_VSMOW_(^17^O/^16^O) of −27.84 ± 0.70‰ for SLAP2 (indicative δ_VSMOW‑SLAP_(^17^O/^16^O) value of–29.68 ± 0.05‰). Therefore, a normalization factor of 1.066 ± 0.027 (*u*_c_; *k* = 1) was applied to report the measured values on the VSMOW-SLAP scale. The intermediate precision (*k* = 1) for VSMOW2 and SLAP2 was 0.51 and 0.47‰ respectively, which was similar to the intermediate precision obtained for δ_VSMOW_(^18^O/^16^O) determinations in these calibration materials.

Analysis of IAEA 604 by ICP-MS/MS gave a δ_VSMOW‑SLAP_(^17^O/^16^O) value of −2.9 ± 0.6‰ (combined uncertainty, *k* = 1), which was not significantly different to the indicative δ_VSMOW‑SLAP_(^17^O/^16^O) value of −3.2 ± 0.4‰^55^ (2-tailed *t* test; *p* = 0.42). Analysis of GRESP by ICP-MS/MS gave a δ_VSMOW‑SLAP_(^17^O/^16^O) value of −13.3 ± 0.6‰ (combined uncertainty, *k* = 1), which was significantly different to the indicative δ_VSMOW‑SLAP_(^17^O/^16^O) value of–17.78 ± 0.02‰^56^ (2-tailed *t* test; *p* = 0.006). The observed bias for GRESP is in line with the bias observed for the δ_VSMOW‑SLAP_(^18^O/^16^O) determinations. It could be reasonably assumed that the determination of δ_VSMOW‑SLAP_(^17^O/^16^O) is likely possible for water materials with low matrix load by the ICP-MS/MS approach. Future studies are required to overcome matrix effects to improve the robustness of δ_VSMOW‑SLAP_(^17^O/^16^O), in addition to δ_VSMOW‑SLAP_(^18^O/^16^O), by this approach.

## Conclusions

This study presents the first successful determination of δ­(^18^O/^16^O) in water using an ICP-MS/MS setup. Moreover, compelling preliminary data demonstrates that δ­(^17^O/^16^O) determinations may also be possible. The application of deuterium reaction gas and measuring in mass-shift mode at +6 amu allowed for the resolution of interferences, especially from the hydrogen-containing polyatomic interferences of ^16^O that interfere on the minor isotopes ^17^O and ^18^O. It is also clear from the level of interferences present that the mass filter in the MS/MS setup is a requirement in order to successfully determine oxygen isotope ratios by quadrupole-based ICP-MS.

The obtained oxygen isotope ratios show low bias from the true value in low matrix water samples. While the overall analytical uncertainty of oxygen isotope ratio measurements using ICP-MS/MS remains higher compared to established reference techniques, the method represents a significant advancement in the field of isotope ratio analysis, and offers potentially greater accessibility particularly to laboratories lacking access to traditional IRMS and CRDS instrumentation. Its operational flexibility and reduced preparation complexity make it a valuable alternative, particularly in applications where large isotopic differences are expected. The ability to perform oxygen isotope analysis and multielemental data using a single analytical platform further enhances its utility. Thus, the work is a strong proof of concept as ICP-MS/MS emerges as a versatile tool for exploratory studies, environmental monitoring, provenance studies and applied geochemistry, where robust and accessible isotope measurements are needed despite slightly higher uncertainties. Further work is required regarding biases from matrix effects, as well as to further lower the sample intake by improving the sample flush time when switching samples, and reduce background oxygen levels by purging the oxygen from the torch box. Moreover, improvements to precision may be possible through further optimizations to the ICP-MS/MS instrument parameters, such as the quadrupole gas cell voltages and through understanding the matrix effects, as well as by developing the methodology further by utilizing modern MC-ICP-MS instrumentation equipped with a collision reaction cell.

## Supplementary Material



## Data Availability

The data underlying this study are available in the published article and its Supporting Information.

## Notes

Spectral interferences and required mass resolutions; ICP-MS/MS operating parameters; results and uncertainties obtained during the validation of the ICP-MS/MS method for δ­(^18^O/^16^O) determinations; results for the multielement analysis of the water materials; results for the analysis of anions in the water materials; elemental cations with excited ionic states close to the ionization energy of oxygen; product ion formation rates in response to reaction cell gas flow rates; single measurement of ^18^O/^16^O depicting the settling and measurement periods; and additional experimental details for multielemental analysis by ICP-MS/MS, ion chromatography measurements, oxygen isotope ratio determination by IRMS and CRDS, and single measurement uncertainty evaluations by ICP-MS/MS (PDF)
