# Supplementary material for: δ(18O/16O) Determinations in Water Using Inductively Coupled Plasma–Tandem Mass Spectrometry
Source: Anal Chem. 2025 Sep 16;97(38):20788–97. doi: 10.1021/acs.analchem.5c02607 (PMC12489894; doi:10.1021/acs.analchem.5c02607)
Supplement: Supplementary file 1 [file ac5c02607_si_001.pdf]

Supporting Information

$\delta(^{18}O/^{16}O)$ determinations in water using inductively coupled plasma – tandem mass spectrometry

Shaun T. Lancaster[1]*, Johanna Irrgeher[1,2], Remi Dallmayr[3], Elisa Conrad[3], Maria Hörhold[3], Pascal Bohleber[3], Melanie Behrens[3], Federica Camin[4], Klara Žagar[5], Polona Vreča[5] and Thomas Prohaska[1,2]

[1] Department of General, Analytical and Physical Chemistry, Chair of General and Analytical Chemistry, Montanuniversität Leoben, 8700 Leoben, Austria.

[2] Department of Physics and Astronomy, University of Calgary, Calgary, Canada.

[3] Alfred Wegener Institute Helmholtz Centre for Polar and Marine Research (AWI), 27570 Bremerhaven, Germany.

[4] Terrestrial Environmental Radiochemistry Laboratory, Division of Physical and Chemical Sciences, Department of Nuclear Sciences and Applications, International Atomic Energy Agency, Vienna International Centre, 1400 Vienna, Austria.

[5] Department of Environmental Sciences, Jožef Stefan Institute, 1000 Ljubljana, Slovenia.

Corresponding author email: shaun.lancaster@unileoben.ac.at

Table of Contents

Table S1: List of interferences for isotopes of oxygen on *m/z* 16, 17, and 18. Mass resolution was calculated from the standard atomic weights[1] and isotopic compositions[2] of the elements. Negative resolutions indicate that the atomic mass of the interfering species is greater than that of the analyte of interest. S3

Table S2: Optimal conditions for measurements made using ICP-MS/MS S4

Table S3: Comparison of measured $\delta_{VSMOW-SLAP}(^{18}O/^{16}O)$ and combined uncertainties between the developed ICP-MS/MS method and the reference techniques for the in-house laboratory reference materials and internationally recognised reference materials S5

Table S4: Results of the multi-element analysis for the in-house laboratory reference materials and internationally recognised reference materials used within this study. S6

Table S5: Results of the anion chromatography analysis for the in-house laboratory reference materials and internationally recognised reference materials used within this study. S7

Table S6: Energy difference (ΔE) between metastable ionic states and the ionisation energy of oxygen (13.62 eV). O ground state: ^3P. O^+ ground state: ^4S. S8

Figure S1: Variation of product ion formation for the reaction of $^{16}O^+$ at different flow rates of (A) hydrogen gas, and (B) deuterium gas. The mass filter (Q1) was fixed at *m/z* 16, and the mass analyser (Q3) monitored the signals on *m/z* 16 – 22. .... S9

Figure S2: Example processed signal obtained for the measured $^{18}O/^{16}O$ of LRM6. Each replicate data point represents the average of 2000 sweeps. The measured $^{18}O/^{16}O$ value of approximately 0.95 results from the tuning of the reaction cell RPa to approximately 500 000 cps for each isotope. S10

Methodology for multi-element determinations S11

Methodology for anion determinations S11

Methodology for comparative analysis of oxygen isotope ratios in water by validated methodologies S12

Single measurement uncertainty assessment S13

References S14

Table S1: List of interferences for isotopes of oxygen on *m/z* 16, 17, and 18. Mass resolution was calculated from the standard atomic weights[1] and isotopic compositions[2] of the elements. Negative resolutions indicate that the atomic mass of the interfering species is greater than that of the analyte of interest.

Analyte	Analyte Abundance (%)	Analyte Atomic Mass (u)	Interfering ion	Interference Abundance (%)	Interference Atomic Mass (u)	Required Mass Resolution (M/dM)
$^{16}O^{+}$	99.757	15.9949	$^{32}S^{2+}$	94.99	15.9860	1801
			$^{15}N^{1}H^{+}$	0.364	16.0079	-1229
			$^{14}N^{2}D^{+}$	0.011	16.0172	-719
$^{17}O^{+}$	0.038	16.9991	$^{34}S^{2+}$	4.25	16.9839	1118
			$^{16}O^{1}H^{+}$	99.746	17.0027	-4712
$^{18}O^{+}$	0.205	17.9992	$^{36}S^{2+}$	0.01	17.9835	1152
			$^{36}Ar^{2+}$	0.3336	17.9838	1170
			$^{17}O^{1}H^{+}$	0.0380	18.0070	-2308
			$^{16}O^{2}D^{+}$	0.0115	18.0090	-1826
			$^{16}O^{1}H_2^{+}$	99.734	18.0106	-1578

Table S2: Optimal conditions for measurements made using ICP-MS/MS

Parameter	Optimisation of signal to background ratio	Isotope ratio determinations	Multi-element determinations	
			ICP multi-element standard solution VI	Silicon
Operating mode	MSMS	MSMS	Q3 Only	MSMS
Cell gas	Deuterium	Deuterium	None	Nitrous oxide
Cell gas flow rate	1 mL min^{-1}	1 mL min^{-1}	N/A	0.5 mL min^{-1}
RPa	^{16}O: 0.0436<br>^{17}O: 0.0333<br>^{18}O: 0.0364	^{16}O: 0.0436<br>^{17}O: 0.0333<br>^{18}O: 0.0364	0	0
RPq	0.45	0.45	0.25	0.45
Sample introduction	Peristaltic pump with 0.38 mm ID pump tubing at 18 rpm.	Peristaltic pump with 0.38 mm ID pump tubing at 18 rpm.	FAST sample introduction system	FAST sample introduction system
Nebulizer	PFA MicroFlow	PFA MicroFlow	PFA MicroFlow	PFA MicroFlow
Spray chamber	Peltier heated SilQ cyclonic spray chamber	Peltier heated SilQ cyclonic spray chamber	Peltier cooled SilQ cyclonic spray chamber	Peltier cooled SilQ cyclonic spray chamber
Spray chamber temperature	50 °C	50 °C	5 °C	5 °C
Interface cones	Nickel	Nickel	Nickel	Nickel
RF power	1600 W	1600 W	1600 W	1600 W
Ar nebulizer gas flow	0.98 L min^{-1}	0.98 L min^{-1}	0.98 L min^{-1}	0.98 L min^{-1}
Ar auxiliary gas flow	1.2 L min^{-1}	1.2 L min^{-1}	1.2 L min^{-1}	1.2 L min^{-1}
Ar plasma gas flow	16 L min^{-1}	16 L min^{-1}	16 L min^{-1}	16 L min^{-1}
Isotope ratio mode	On	On	Off	Off
Dwell time	50 ms	2 ms	50 ms	50 ms

Table S3: Comparison of measured $\delta_{VSMOW-SLAP}(^{18}O/^{16}O)$ and combined uncertainties between the developed ICP-MS/MS method and the reference techniques for the in-house laboratory reference materials and internationally recognised reference materials.

**Material**	**Reference Techniques**		**ICP-MS/MS**		**Bias**
	**$\delta_{VSMOW-SLAP}(^{18}O/^{16}O)$ (‰)**	**u_c (k = 1) (‰)**	**$\delta_{VSMOW-SLAP}(^{18}O/^{16}O)$ (‰)**	**u_c (k = 1, n = 3) (‰)**	**$\Delta(^{18}O/^{16}O)$ (‰)**
GRESP	-33.3	0.04	-22.4	2.66	+10.9
IAEA 604	-5.86	0.04	-6.58	1.09	-0.72
LRM 1	0.36	0.04	-11.6	0.79	-11.9
LRM 2	-9.12	0.03	-9.69	0.49	-0.57
LRM 3	-18.91	0.03	-18.7	0.92	+0.18
LRM 4	-54.03	0.03	-55.1	0.64	-1.08
LRM 5	0.4	0.03	-0.15	0.72	-0.55
LRM 6	-10.77	0.1	-11.8	0.48	-0.99
LRM 7	-3.22	0.1	-3.79	0.88	-0.57
LRM 8	-7.34	0.1	-13.2	0.97	-5.89
LRM 9	-26.64	0.1	-30.0	0.68	-3.40
LRM 10	-42.395	0.02	-42.2	0.68	+0.20
LRM 11	-53.005	0.03	-53.8	0.73	-0.79

Table S4: Results of the multi-element analysis for the in-house laboratory reference materials and internationally recognised reference materials used within this study.

Material	Measured Mass Concentration (ng mL^{-1})													
	**Na**	**Mg**	**Al**	**Si**	**K**	**Ca**	**V**	**Cr**	**Mn**	**Fe**	**Co**	**Ni**	**Cu**	**Zn**
VSMOW2	677	9.79	7.11	n.d.	12.9	280	0.113	0.111	8.24	0.657	0.466	23.3	4.60	15.9
SLAP2	1 120	40.0	19.7	n.d.	19.1	276	0.120	0.120	3.01	0.764	0.165	3.28	6.24	13.4
GRESP	9 580	6.96	1 940	20 300	484	394	0.231	0.662	8.62	5.37	0.130	0.489	8.79	<LOD
IAEA 604	2 430	550	44.2	4 090	202	194	0.020	0.470	16.9	<LOD	0.200	10.1	22.8	41.3
LRM 1	4 170	624	7.68	349	249	645	9.06	131	109	485	9.50	254	5.00	34.9
LRM 2	2.86	1.45	3.91	<LOD	40.1	517	6.91	4.74	31.6	29.6	6.20	3.71	0.373	38.6
LRM 3	431	23.5	4.36	<LOD	225	707	6.93	6.52	15.1	57.7	6.37	6.93	0.861	66.8
LRM 4	910	24.0	74.7	1 360	<LOD	5 120	69.1	48.8	72.2	452	59.7	<LOD	8.97	505
LRM 5	1 700	42.5	6.07	1 413	62.3	387	0.159	0.327	31.3	2.18	1.42	68.1	39.4	41.0
LRM 6	440	0.197	0.437	<LOD	429	51.3	0.697	0.485	0.710	3.70	0.598	0.003	0.055	3.34
LRM 7	59.7	0.501	16.3	<LOD	11.6	60.9	0.691	0.496	0.788	3.90	0.604	<LOD	0.054	3.39
LRM 8	18 400	21.4	39.1	89 600	3 890	15 600	69.5	46.5	70.4	210	59.7	<LOD	5.02	345
LRM 9	2 230	1 040	3.23	6 220	410	1 360	<LOD	1.89	<LOD	2.22	0.017	0.231	2.20	1.34
LRM 10	518	402	0.434	139	114	152	<LOD	1.55	<LOD	<LOD	0.031	0.214	12.2	8.14
LRM 11	2 670	454	26.7	2 990	406	1 500	<LOD	0.347	<LOD	5.66	0.023	0.326	2.03	7.53

n.d. = not determined due to insufficient sample volume

Table S5: Results of the anion chromatography analysis for the in-house laboratory reference materials and internationally recognised reference materials used within this study.

**Material**	**Measured Mass Concentration (ng mL^{-1})**					
	**Fluoride**	**Chloride**	**Nitride**	**Bromide**	**Nitrate**	**Sulphate**
VSMOW2	329	<LOD	<LOD	<LOD	<LOD	<LOD
SLAP2	394	<LOD	<LOD	<LOD	214	211
GRESP	498	<LOD	<LOD	<LOD	283	<LOD
IAEA 604	647	<LOD	<LOD	<LOD	342	175
LRM 1	<LOD	130 000	423	847	<LOD	1 140
LRM 2	<LOD	<LOD	<LOD	<LOD	<LOD	<LOD
LRM 3	<LOD	517	<LOD	<LOD	627	249
LRM 4	<LOD	543	<LOD	<LOD	299	1 360
LRM 5	<LOD	<LOD	<LOD	<LOD	516	<LOD
LRM 6	<LOD	<LOD	<LOD	<LOD	117	<LOD
LRM 7	<LOD	<LOD	<LOD	<LOD	94.0	<LOD
LRM 8	<LOD	163	384	<LOD	233	1 015
LRM 9	<LOD	385	<LOD	<LOD	117	185
LRM 10	<LOD	<LOD	<LOD	<LOD	<LOD	<LOD
LRM 11	<LOD	864	176	<LOD	<LOD	203

Table S6: Energy difference (Δ*E*) between metastable ionic states and the ionisation energy of oxygen (13.62 eV). O ground state: ^3P. O^+ ground state: ^4S.

Matrix ion	Ion ground state term	Ion energy level(s) closest to the ionization energy of oxygen			Δ*E* (eV)
		Term	J	Energy (eV)	
C^+	^2P	^2P	3/2, 1/2	11.26	-2.36
Si^+	^2P	^4P	5/2, 3/2, 1/2	13.48	-0.14
Si^+	^2P	^2D	5/2, 3/2	15.01	1.39
P^+	^3P	^1S	0	13.16	-0.46
S^+	^4S	^2P	3/2, 1/2	13.40	-0.22
Cl^+	^3P	^3P	1, 0	13.04	-0.58
Cl^+	^3P	^1D	2	14.41	0.79

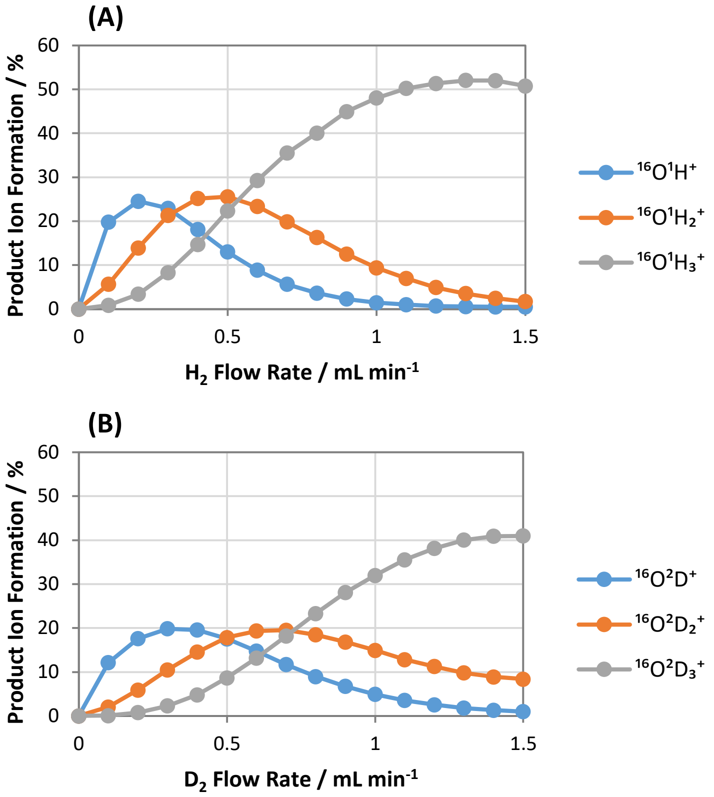

Figure S1: Variation of product ion formation for the reaction of $^{16}O^{+}$ at different flow rates of (A) hydrogen gas, and (B) deuterium gas. The mass filter (Q1) was fixed at *m*/*z* 16, and the mass analyser (Q3) monitored the signals on *m*/*z* 16 – 22.

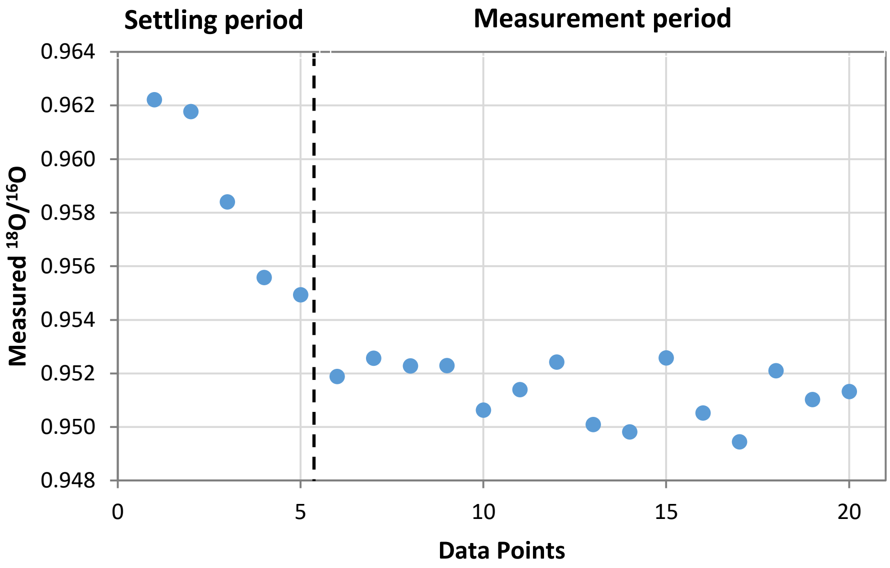

Figure S2: Example processed signal obtained for the measured $^{18}O/^{16}O$ of LRM6. Each replicate data point represents the average of 2000 sweeps. The measured $^{18}O/^{16}O$ value of approximately 0.95 results from the tuning of the reaction cell RPa to approximately 500 000 cps for each isotope.

Methodology for multi-element determinations

Chemicals and standards

ICP multi-element standard solution VI (Certipur; Merck, Darmstadt, Germany) and silicon (1000 µg mL^{-1}; SPEX Chemicals, Metuchen, NJ, USA) were used as a calibration standard for multi-element analysis of all water materials used in this study. Nitric acid (HNO_3, w = 65%, p.a. grade; Carl Roth GmbH, Karlsruhe, Germany) was purified using a sub-boiling distillation system (Savillex DST-4000, AHF Analysentechnik, Tübingen, Germany) and utilized for acidification of the water materials for multi-element analysis. An indium single element ICP-MS standard (γ = 1000 µg mL^{-1}; Certipur, Merck) diluted to 650 ng L^{-1} in dilute HNO_3 (w = 2%) was used as an internal standard for multi-element analysis.

ICP-MS/MS analysis of multi-elemental content in water standards and internationally recognised reference materials

Multi-element determinations were carried out using a NexION 5000 (PerkinElmer, Waltham, MA, USA). All water LRMs and reference materials were acidified with HNO_3 (final HNO_3 mass fraction of w = 2%) before analysis. Further dilutions (if necessary) were made using w = 2% HNO_3. The materials were analysed primarily for quantities of Na, Mg, Al, K, Ca, V, Cr, Mn, Fe, Co, Ni, Cu, and Zn. The PerkinElmer NexION 5000 ICP-MS/MS was operated in standard mode, with no cell gas applied (Table S2). Silicon was measured using nitrous oxide (medicinal grade; Linde Gas GmbH, Stadl-Paura, Austria) as a reaction gas for interference removal. Q1 was set to m/z 29, and Q3 was set to m/z 61 (SiO_2^+ product ion).

Methodology for anion determinations

Chemicals and standards

Standard solutions of fluoride, chloride, bromide, nitrate, nitride, and sulphur (1000 µg mL^{-1}; VWR Chemicals, Slolon, OH, USA) diluted in reagent grade I water (18.2 MΩ cm; MilliQ IQ 7000, Merck-Millipore, Darmstadt, Germany) were used for calibration. The mobile phase was comprised of a mixture of 1.0 mmol L^{-1} sodium bicarbonate and 3.2 mmol L^{-1} sodium carbonate prepared in reagent grade I water. Concentrated sulphuric acid (96%, p.a. grade; Carl Roth GmbH, Karlsruhe, Germany) diluted in reagent grade I water to 0.1 mol L^{-1} was used as a suppressor solution.

Ion chromatography analysis of the anion content in water standards and internationally recognised reference materials

A Metrohm Eco IC system (Metrohm, Herisau, Switzerland) was used for the determination of anions in the water mateerials. Samples were loaded onto a 10 µm sample loop and injected onto a Metrosep A Supp 17 (150 mm) separation column. The mobile phase flow rate was 0.8 mL min^{-1}. The sample then passed through a suppressor column to suppress the carbonate background, and detected using a built in conductivity detector. The analyte peaks were integrated and the concentrations calculated using Metrohm MagIC v3.3.

Methodology for comparative analysis of oxygen isotope ratios in water by validated methodologies

Isotope ratio mass spectrometry (IRMS)

Calibration of LRM1 – LRM4 for isotopic composition of oxygen was performed by using the CO_2-H_2O equilibration technique.[3,4] Measurements were conducted on a dual inlet isotope ratio mass spectrometer (DI IRMS, Finnigan MAT DELTA plus, Finnigan MAT GmbH, Bremen, Germany) with an automated CO_2-H_2O and H_2-H_2O HDOeq 48 Equilibration Unit and a water bath temperature of 18 °C. The water vapour trap was cooled to −55 °C. CO_2 gas was used as working standard for equilibration of water and equilibration of CO_2-H_2O was for 6 hours. The results are expressed in standard delta notation (equation 1), given in per mil (‰) deviation of the sample from the standard. Measurements were carried out together with VSMOW2 and SLAP2 calibration standards. Results were normalised to the VSMOW-SLAP scale using two point normalisation, and the uncertainties of the LRMs were calculated using the Kragten method.[5]

Cavity ring-down spectroscopy (CRDS)

The value of LRM5 came from analysis of 415 units performed in IAEA by using IRMS (Finnigan Delta+) and 2 Picarro CRDS instruments ((Picarro L1102-i and L2130-i) via autosampler syringe-controlled injection of 2 µL of sample. VSMOW2 and SLAP2 were used as calibrants to ensure traceability to VSMOW-SLAP scale and GISP was used as quality control sample. The assigned value and uncertainty were calculated using the Excel macro-spreadsheet based SICalib calibration program[6], available for free download. Memory and drift corrections for all measurements were applied. Combined standard uncertainty included measurements uncertainty, weighted uncertainty of weighted mean values, bias between instruments and assigned uncertainty of the calibrants.

LRM6 – LRM9 were calibrated using a Picarro CRDS instrument (model L2140-i) equipped with an autosampler and a vaporizer for liquid water sample analysis. The measurement and correction scheme described by *Van Geldern and Barth*[7] was followed. Each unknown sample was measured as two separate samples with 4 injections each. Two in-house standards were used during calibration to the VSMOW-SLAP scale; one quality control standard (measured as a sample), and one drift correction standard measured four times during the run. At the beginning of each acquisition, the in-house standard injections are also used to determine the memory effect, which results in the estimation of the sample carry-over from injection to injection. The combined measurement uncertainty was calculated from (1) long-term stability (root mean square error of replicate measurements of the quality control standard over approximately one year), (2) the offset of the quality control standard to its reference value, and (3) the uncertainty of the reference standards. The uncertainty components were combined using the root-sum-square.[8]

LRM 10 and LRM 11 are present Alfred Wegener Institute (AWI) in-house standards and are calibrated against the reference materials VSMOW2 and SLAP2 yearly. Measurements, corrections and calculation of the combined uncertainty for the in-house standards was done following the IAEA recommendations using the SICalib software.[6]

Single measurement uncertainty assessment

Evaluation procedure

Although the work within this study focussed primarily on intermediate precision, indicative ranges for combined uncertainties of single measurements have also been evaluated. For the standard sample bracketing approach, the single measurement uncertainty is calculated from the uncertainty in the sample measurement and the two bracketing standard measurements, as well as analysis of the two calibration standards.

In the stable isotope ratio community, there are currently two approaches to handling the uncertainty components from the sample and the two bracketing standards to calculate the single measurement uncertainty. The most common approach is to use the single measurement repeatability (calculated using the standard error of the mean of a measurement ($s/\sqrt{n}$)).[9,10] However, this approach has been disputed, noting that data points generated in a single measurement cannot be considered independent and therefore only the standard deviation (s) can be used.[11] In the interest of transparency, both approaches for single measurement uncertainty have been included in this work.

Single measurement uncertainties for $\delta_{VSMOW\text{-}SLAP}(^{18}O/^{16}O)$

Following the consideration that single measurement standard error of the mean of the sample and bracketing standard should be used[9,10], the obtained combined uncertainties ranged from 0.56 ‰ to 1.08 ‰, with a median uncertainty of 0.72 ‰. In this case, the highest contribution to the uncertainty budget came from the measurement of the sample and the replicate measurement of VSMOW2 calibration standard.

Following the consideration that single measurement standard deviations of the sample and bracketing standard should be used[11], the obtained combined uncertainties obtained ranged from 1.40 ‰ to 3.86 ‰, with a median uncertainty of 2.20 ‰. Here, the highest uncertainty contributions originated from the measurement of the sample and the two measurements of the bracketing standard.

References

(1) Prohaska, T.; Irrgeher, J.; Benefield, J.; Böhlke, J. K.; Chesson, L. A.; Coplen, T. B.; Ding, T.; Dunn, P. J. H.; Gröning, M.; Holden, N. E.; Meijer, H. A. J.; Moossen, H.; Possolo, A.; Takahashi, Y.; Vogl, J.; Walczyk, T.; Wang, J.; Wieser, M. E.; Yoneda, S.; Zhu, X. K.; Meija, J. Standard Atomic Weights of the Elements 2021 (IUPAC Technical Report). *Pure Appl. Chem.* **2022**, *94* (5), 573–600. https://doi.org/10.1515/pac-2019-0603.

(2) Meija, J.; Coplen, T. B.; Berglund, M.; Brand, W. A.; De Bièvre, P.; Gröning, M.; Holden, N. E.; Irrgeher, J.; Loss, R. D.; Walczyk, T.; Prohaska, T. Isotopic Compositions of the Elements 2013 (IUPAC Technical Report). *Pure Appl. Chem.* **2016**, *88* (3), 293–306. https://doi.org/10.1515/pac-2015-0503.

(3) Epstein, S.; Mayeda, T. Variation of O18 Content of Waters from Natural Sources. *Geochim. Cosmochim. Acta* **1953**, *4* (5), 213–224. https://doi.org/10.1016/0016-7037(53)90051-9.

(4) Avak, H.; Brand, W. A. The Finning MAT HDO-Equilibration - A Fully Automated H2O/Gas Phase Equilibration System for Hydrogen and Oxygen Isotope Analyses. *Thermo Electron. Corp. Appl. News* **1995**, *11*, 1–13.

(5) *Good Practice Guide for Isotope Ratio Mass Spectrometry*, 1st ed.; Carter, J. F., Barwick, V. J., Eds.; FIRMS: Bristol, UK, 2011.

(6) Gröning, M. Improved Water Δ2H and Δ18O Calibration and Calculation of Measurement Uncertainty Using a Simple Software Tool. *Rapid Commun. Mass Spectrom.* **2011**, *25* (19), 2711–2720. https://doi.org/10.1002/rcm.5074.

(7) Van Geldern, R.; Barth, J. A. C. Optimization of Instrument Setup and Post-Run Corrections for Oxygen and Hydrogen Stable Isotope Measurements of Water by Isotope Ratio Infrared Spectroscopy (IRIS). *Limnol. Oceanogr. Methods* **2012**, *10* (DECEMBER), 1024–1036. https://doi.org/10.4319/lom.2012.10.1024.

(8) Magnusson, B.; Näykki, T.; Hovind, H.; Krysell, M.; Sahlin, E. *Handbook for Calculation of Measurement Uncertainty in Environmental Laboratories, Nordtest Report TR 537*, Fourth Edi.; 2017.

(9) Vogl, J.; Rosner, M.; Kasemann, S. A.; Kraft, R.; Meixner, A.; Noordmann, J.; Rabb, S.; Rienitz, O.; Schuessler, J. A.; Tatzel, M.; Vocke, R. D. Intercalibration of Mg Isotope Delta Scales and Realisation of SI Traceability for Mg Isotope Amount Ratios and Isotope Delta Values. *Geostand. Geoanalytical Res.* **2020**, *44* (3), 439–457. https://doi.org/10.1111/ggr.12327.

(10) Vogl, J.; Rienitz, O.; Pramann, A.; Flierl, L. Scale Conversion and Uncertainty Calculations in Isotope Delta Measurements. *Geostand. Geoanalytical Res.* **2022**, *46* (4), 773–787. https://doi.org/10.1111/ggr.12450.

(11) Horsky, M.; Irrgeher, J.; Prohaska, T. Evaluation Strategies and Uncertainty Calculation of Isotope Amount Ratios Measured by MC ICP-MS on the Example of Sr. *Anal. Bioanal. Chem.* **2016**, *408* (2), 351–367. https://doi.org/10.1007/s00216-015-9003-9.
